# O.2.1-4 Physical activity program for people with intellectual disability: results from an implementation study

**DOI:** 10.1093/eurpub/ckad133.109

**Published:** 2023-09-11

**Authors:** Marieke Wouters, Alyt Oppewal, Dederieke Maes-Festen

**Affiliations:** Erasmus Medical Center, Rotterdam, The Netherlands; m.wouters.2@erasmusmc.nl; Erasmus Medical Center, Rotterdam, The Netherlands; m.wouters.2@erasmusmc.nl; Erasmus Medical Center, Rotterdam, The Netherlands; m.wouters.2@erasmusmc.nl

## Abstract

**Purpose:**

Many people with an intellectual disability (ID) are inactive and have low physical fitness. A physical activity (PA) program was specially developed for this group and found to be effective to increase PA and preventing or delaying deterioration in physical fitness. However, implementation in practice has not yet been successful. The aim of this project is to study the implementation of this PA program, to be able to increase successful implementation and scaling up in the future.

**Methods:**

we are systematically implementing the PA program on three sites within the care for people with ID in the Netherlands. The key elements of the PA program are: three times a week for 45-60 minutes; training in a group; focus on increasing endurance, strength, balance and flexibility; increasing intensity and duration; under supervision of a PA expert; including education on healthy PA. Implementation is guided by implementation support practitioners, who are using the Grol and Wensing implementation model as a guide for the implementation. Implementation is studied by collecting data on i.a. acceptability, feasibility, fidelity, and determinants on three moments (three months after start of implementation process, one and six months after start of the PA program). This was done by interviewing the implementation support practitioners and the staff implementing the PA program on site. Furthermore, field notes and logs are collected. Interview data are verbally transcribed and coded. All data are thematically analyzed, both inductive and deductive.

**Results:**

Four to five months after start of the implementation process, the PA program started. Preliminary results of the first data collection show that acceptability, feasibility and fidelity of the program is moderate. Not all key elements are implemented as prescribed, like frequency and the way of involvement of the PA expert. Suggestions are made to improve the chance of successful implementation of the PA program.

**Conclusions:**

Some of the original key elements and preconditions should be reconsidered to make the program more suitable for further implementation and scaling up within the care for people with an ID.

**Funding:**

This study was funded by ZonMW.

